# Prevalence and associated risk factors of Intestinal parasites in rural high-mountain communities of the Valle del Cauca—Colombia

**DOI:** 10.1371/journal.pntd.0008734

**Published:** 2020-10-09

**Authors:** Magda Gileydi Peña-Quistial, Javier Antonio Benavides-Montaño, Nestor Javier Roncancio Duque, Gerardo Alejandro Benavides-Montaño

**Affiliations:** 1 Animal Science Department, Universidad Nacional de Colombia, Palmira Valle, Colombia; 2 Laboratorio clínico Colcan—Sede Bucaramanga, Colombia; Universidade Federal de Minas Gerais, BRAZIL

## Abstract

The aim of this study was to measure the prevalence of gastrointestinal parasites (GI) in domestic animals and children in high mountain populations in the districts of Combia and Toche, Valle del Cauca–Colombia. These communities have been affected by the armed conflict in Colombia and are susceptible to health risk factors related to the Colombian post-conflict. Prevalence and risk factors were measured using Bayesian methods on 45 structured interviews applied to 29 families in Combia and 16 in Toche. This inquire aimed to analyze the socio-economic and demographic factors associated with the presence of parasites. This interview was conducted with 50 children: 40 (80%) from Rita Sabogal school district of Toche, and 10 (20%) from Tablones—Atanasio Girardot schools. 23 faecal samples from asymtomatic children from these schools were collected. Subsequently, 308 animals were characterized through the analysis of 64 faecal samples from asymptomatic individuals (20,8%); 18/41 from dogs (43,9%), 18/175 from poultry (10,3%), 7/13 from cats 56,84%, 6/20 from equines (30%) and from 15/59 cattle (25,43%). The prevalence of intestinal parasites among children under six years was 60% [95% PI = 41%-78%]; *Endolimax nana*, 24% [95% PI = 9,8%-42%]; *Iodamoeba buetschlii*, 16% [95% PI = 4,7%-32%]; *Entamoeba coli*, 35% [95% PI = 18%-55%]; *Giardia lamblia*, 12% [95% PI = 2,7%-27%]. In Equids the presence of *Strongylus* spp was 37% [95% PI = 10%-71%]; *Parascaris equorum*, 37% [95% PI = 10%-71%]; in dogs, *Dipylidium caninum* was 20% [95% PI = 6%-39%]; *Trichuris trichiura*, 9% [95% PI = 1,3%-26%]; *Toxocara canis*, 25% [95% PI = 9%-46%]; in cats, *Toxocara cati* had a prevalence of 44% [95% PI = 16%-75%]; cyst of *Eimeria* spp, 15% [95% PI = 3,4%-33%]; in poultry and *Eimeria zuernii* in cattle, 50% [95% PI = 23%-77%]. There was no association with exposure of humans to animal parasites. However, we conclude that female and children under 6 years of age are more likely OR (6,72–2,3) to get parasites.

## Author summary

The Colombian armed conflict affected vulnerable communities with already precarious governmental and health care assistance. Epidemiological surveillance applied to public health is fundamental to prevent zoonotic diseases in vulnerable Colombian populations. Systematic analysis are needed in order to diagnose the problem, to design, to implement public health practices and to evaluate the interventions associated with the identification of parasitic risk factors. In livestock there are several risks linked to human behaviors that have an impact on the occurrence, dissemination, perpetuity of parasitic agents and the quality of life of people. Therefore, it is required to study the risk of parasitic diseases for the farmer production at the high mountain rural communities of Palmira in the post-conflict period. Overall, this study represents an important approach to understand endemic parasites in high elevations of the Andes region of Colombia, to prevent zoonotic diseases and to recognize the relevance of the One Health approach considering ecosystems, domestic animals, wild animals and human health as a unit.

## Introduction

There are several Colombian territories with difficult access that have not been intervened by the health services after the implementation of the Colombian peace agreement and the National Rural Health Plan (PNSR) [1,2]. Recently, significant advances have been made studying vulnerable rural-populations and how the displacement generated by the Colombian armed conflict has affected the life settings of these communities [3–6]. Populations with low quality of health, constant migration and displacement suffer serious parasitic diseases acting as temporal or permanent carriers, disseminating intestinal parasites [7,8]. Parasites such as helminths and protozoa are the most frequent, affecting more than 2 billion people worldwide, reaching a high prevalence in some regions [9]. Previous studies conducted in Colombia indicate that human communities affected by forced displacement are exposed to *Giardia* spp with a prevalence of 15% to 60,4% [10]. The presence of these parasites is associated with the use of communal toilets, and the provision of water by municipal pipelines and by individual tanks; however, it is mostly linked to the poor water quality control in aqueduct and agricultural labor [11,12].

This study aimed to determine the prevalence of parasites in domestic animals and children populations subject to migration and forced displacement in high mountain areas of Colombia.

## Methods

### Ethics statement

The study was approved by the Ethical Committee Board IDEA-098-18 assigned by the Institute of Environmental Studies. During the project “School of Permanent Environmental University Thinking. The Post Agreement as an Opportunity for a New Relationship between Society and its Natural and Cultural Heritage”. This project implied little to no risk for the participants. Different meetings with the communities of Toche and Combia were held in order to clarify the aims and study protocol. Informed consent was obtained from parents and teachers before sample collection. In the case of the pet samples; the owners provided written consent for sample collection.

### Study area and population

**Fig 1:**
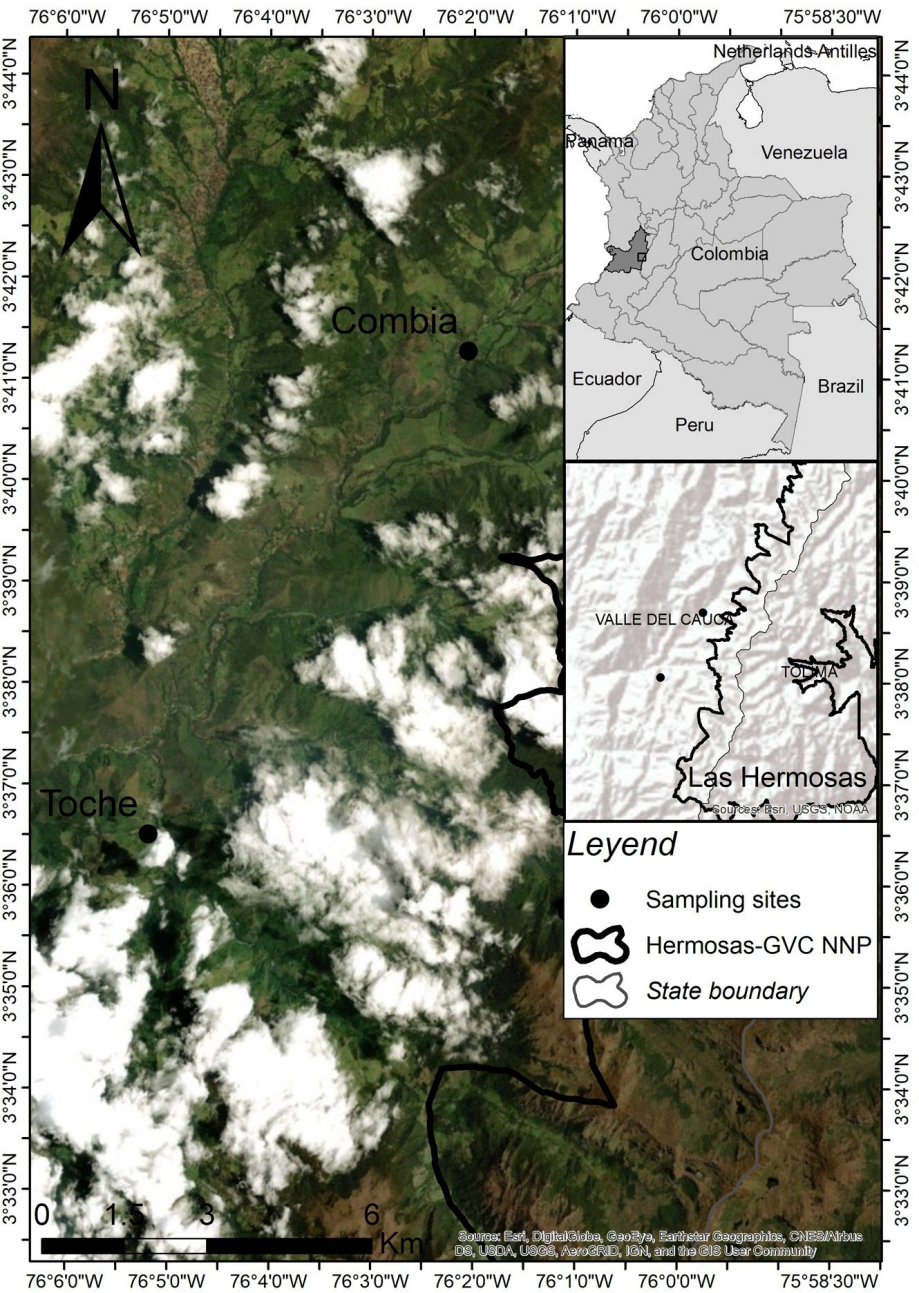
The geographic location of the district of Toche and Combia, located among 1600 to 4100 m.a.s.l. Palmira, Valle del Cauca—Colombia.

The field study was carried out in the department of Valle del Cauca, in the rural area of Palmira, in the districts of Combia (lat: 3.687862, long: -76.034223, alt:2179.9) and Toche (lat: 3.608412, long: -76.086415 alt: 1628.59) between April 27^th^ and December 5th, 2019 (Fig 1).

### Type of study

The present study is a cross-sectional observational type of prevalence that allows to evaluate the relationship between a disease or a health-related characteristic and other variables of interest existing in a specific population and defined moment. The presence or absence of the disease and/or its variables are assessed in a sample and at a particular time, without allowing the temporal sequence of cause and effect to be evaluated [13]. The prevalence of gastrointestinal parasites was estimated using Bayesian methods employing an uninformative *a priori* binomial distribution (between 0 and 100%) and assuming that the posterior distributions were fitted to a uniform distribution [14,15].

### Sample

45 structured interviews were applied to 29 families in Combia and 16 families in Toche in order to analyze the socio-economic and demographic factors associated with the parasitic presence. These interviews represent the asymptomatic children studied, a total of 50 asymtomatic children, 40 (80%) from Rita Sabogal school district of Toche, and 10 (20%) from Tablones—Atanasio Girardot schools, among which were collected 23 faecal samples which represents 46% of the total population of children. A total of 308 animals were characterized analyzing 64 faecal samples from asymptomatic individuals (20,8%), 18/41 from dogs (43,9%), 18/175 from poultry (10,3%), 7/13 from cats 53,84%, 6/20 from equines (30%) and 15/59 from cattle (25,42%).

The data obtained was related to different risk variables or exposure factors such as hygiene habits, living with pets (dogs and cats), source of the river and quality of water (river, stream, and water aqueduct); or socio-economic factors, such as schooling level (primary and secondary), income and occupation of the inhabitants. Stool samples were obtained from children aged between 4 months to 12 years of age. Samples were collected from the rectum. The parasite analysis was performed by direct microscopic examination, using a microscope ZEISS AxioCam ICc 1 and with flotation with Sheather technique, fixation and coloring techniques of Zieh Nielsen, Lugol, physiological saline as well as kato-katz method [16–18].

### Survey validation

The survey was validated by several professionals, considering the relevance and sufficiency of each item, making the corresponding adjustments to the original version. During the second phase, the test was validated in Combia and Toche. Upon completion of this phase, a descriptive analysis was carried out using frequency tables and statistical indicators of focus and dispersion according to previously adopted methodologies [19].

### Relationship of the explanatory variables with presence of gastrointestinal parasites

The relationship of the presence of gastrointestinal parasites and the quantitative explanatory variables were measured by logistic regression methods and the qualitative explanatory variables were studied with frequency analyses (contingence tables). First, we carried out a correlation test between quantitative explanatory variables in order to eliminate collinearity (autocorrelation) [20]. The Spearman coefficient of correlation was used to evaluate this association (no data distributed normally, Shapiro-Wilk test: W = 0.90, P = 0.04). When two variables were associated (Spearman rank correlation coefficient: *P*-r^s^ <0.05), one of them was eliminated [21]. The selected final variables are those that were not associated with others and thus have the least number of explanatory variables for running the regression analysis. As a result, six out of the 25 were considered to be explanatory variables (age, education level of the carrier, garbage dumps presence, handwashing practices during meal preparation, deworming, pet contact). Given that the presence of parasites is a presence/absence variable, the relationships were evaluated using logistic regression. This was done by means of a Bayesian approach and using uninformative prior distribution to the precision of the explanatory variable effect, and uninformative prior distributions to the intercept (alpha) and slopes (beta). It was assumed that the posterior distributions of the intercept and the slope of each variable had a normal distribution [14,15]. The interaction (multiplicative effects) between the explanatory variables we are not taken into account. With the purpose of selecting the best model, the Deviance Information Criterion (DIC) was employed. Initially, the model was run with all the possible explanatory variables; afterwards, the DIC was estimated. Later, one variable was extracted, the model was run and the DIC was assessed once again. When the DIC was higher, the variable was returned and another one was extracted. When the DIC resulted lower, that variable was removed definitively and an additional one was added, until we got the model with the lowest DIC. The relationship with the qualitative variables was evaluated for each one of seven variables with contingence tables using the Bayesian approach. Estimation of the prevalence, interception and slopes of logistic regression and the Odds values to the contingence tables was done using Markov chains with 100.000 interactions and taking account from the 10.001 iteration to the final estimation. The analyses were performed using OpenBugs 3.2.2 software [22].

### Application of the data collection instrument (Survey)

After the validation process was completed, the surveys were structured in 51 questions with an application time of 15–20 minutes. The questions were distributed in three sessions. The first one inquired about the personal data and sociodemographic profile of the adult informants: this session presented nine items that record sex, age, ethnicity, highest educational level, marital status, last occupation, promoter health entity, (Entidades Promotoras de Salud-EPS-), regime or type of affiliation to the health system (contributory for people with employment, payment capacity–such as formal and independent workers, pensioners and their families, subsidy for vulnerable population, unable to pay); the second one examined the household socio-economic profile. This session featured three items that evaluated the socio-economic stratum of housing, income, type of housing, location and health status; and third: farming systems, type of areas destined to the production system, vaccination plans and deworming.

## Results

Toche and Combia are migrant populations engaged in livestock and vegetable agriculture. These populations have suffered forced displacement [23]. Regarding basic sanitation, we find that currently its inhabitants are supplied with surface water and there is no sewage system, which increases the probability (OR 1,5) of parasitic exposure when making grey and excreted open water discharges in sources of surface waters. There are communal septic tanks in the inhabited areas, and rural aqueducts without adequate water treatment for consumption. It is a high environmental impact area, associated with burning trees, inappropriate use of soils and forests, overgrazing of cattle on high-grade slopes and an inadequate planting system with the use of artificial pesticides and herbicides previously reported [24]. Regarding the Educational System, Combia and Toche, has two schools: Atanasio Girardot with 10 (elementary) students, and Rita Sabogal with 40 (elementary and high school) students. The populations of these areas are transitory, with a low level of education, few job opportunities (housewives, catch crops) and recreation, low conception, low birth rates and high cohabitation with dogs (86,4%) and cats (54,5%) (2 to 3 animals per household). People in this area relate to dogs and cats in a dominionistic way, giving them value in accordance to what they provide to them, such as protection in the case of dogs and rodent control for cats. Most of the time dogs and cats are kept outdoors and not allowed in the child’s room [25].

**Fig 2:**
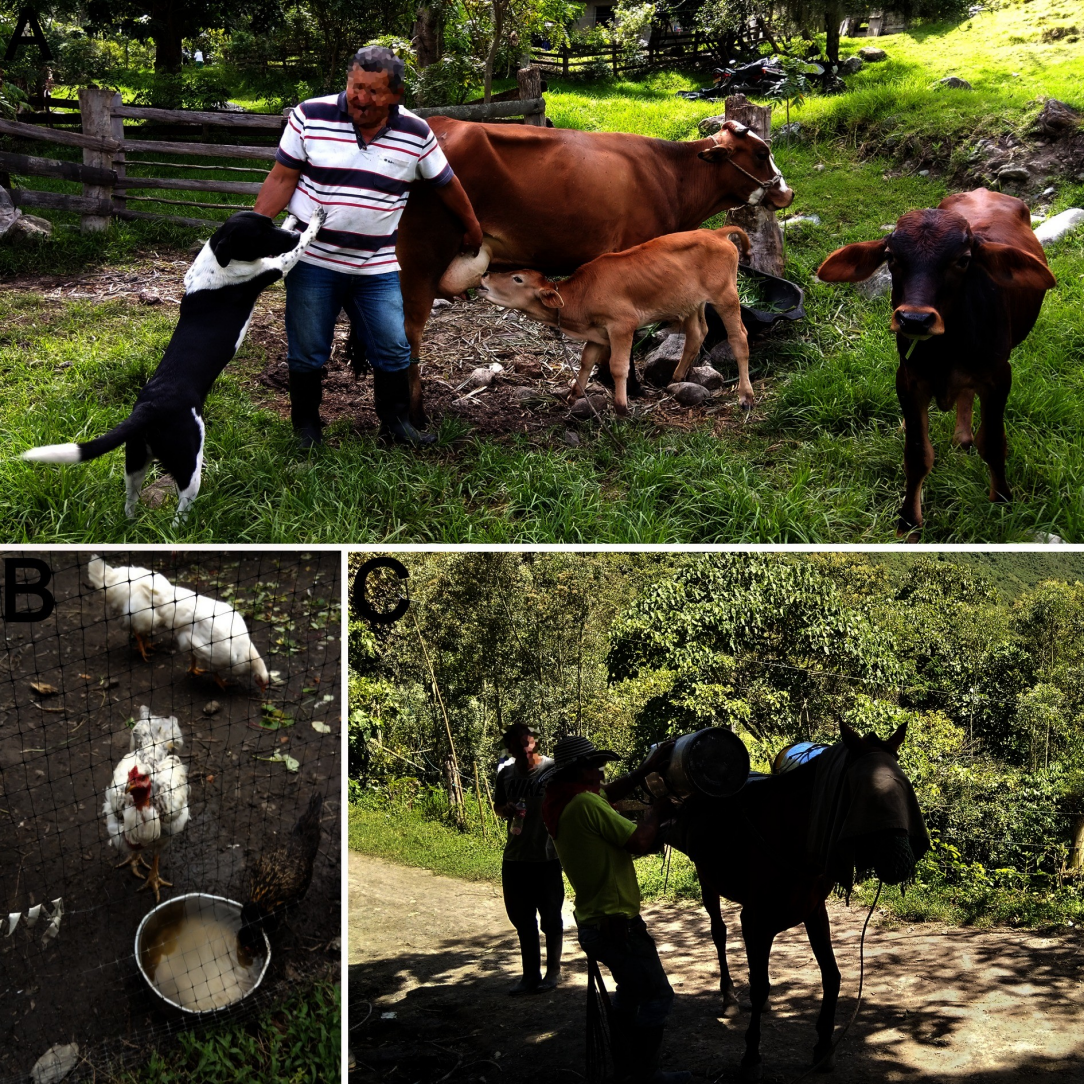
Peasant production systems of Toche and Combia. (A) Small milk producers with their pets. (B) Creole backyard chicken system, a potential resource seldom exploited by housewives. (C) The horse is the conveyance to transport the milk from high mountain areas to the collection sites in Combia and Toche.

**Fig 3:**
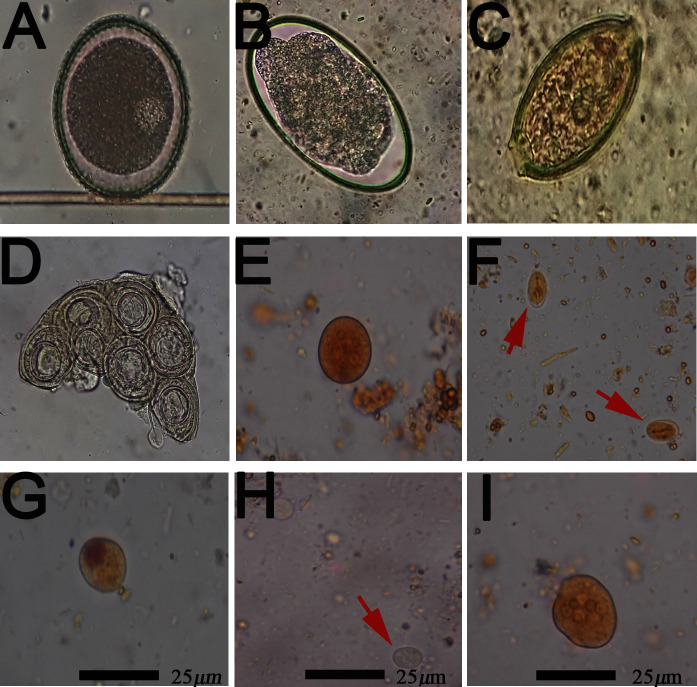
Parasites of children and pets in Combia and Toche. (A) *Toxocara canis* (B) *Strongylus* spp (C) *Trichuris trichiura* (D) Egg packet *Dipylidium caninum* (E) Cyst of *Entamoeba coli* 40x (F) Cyst *Giardia lamblia* 40x (G) Cyst of *I*. *buetschlii* 40x (H) Cyst of *Endolimax nana* 40x (I) *Entamoeba coli* 40x.

Dairy and dual-purpose livestock represents 19,2% of the animal population. It is a small production system, with less than 10 animals per owner, an artisanal subsistence production that guarantees food security that revolves around poultry, eggs, milk and meat (Fig 2A, 2B and Fig 2C). The population of equidae (mules and horses) is 6,5%, 3–6 animals per owner. These animals fulfill sustenance and transport functions, allowing milk and farm products to be moved from high mountain areas (Fig 2C). Backyard poultry production is representative, at a percentage of 56,81%, less than 30 birds (posture and fattening) for self-consumption (Fig 2B). Other activities such as cuniculture, fish farming and beekeeping are rarely employed in these territories. Communities have a low level of medical and hospital assistance (every month). Their economic activity is focused on the cultivation of vegetables: (cabbage, onion, parsley, coriander), bread products (beans, potatoes, bananas, cassava) and the gardening of fruit trees (blackberry, peach, apple, pear, avocado and orange). From 87 coprological samples that were analyzed (23 from children and 64 from animals), children under 6 years old had a prevalence of 60%, SD±9,6%, (n = 23), IC95% (41%-78%). The most common parasites were amoeba’s type *Endolimax nana* (24%), *Iodamoeba buetschlii* (16%) and *Entamoeba coli* (35%), flagellates, *Giardia lamblia* (12%) (Table 1, Table 2, Fig 3). Horses were positive for the presence of *Strongylus* spp (37%), *Parascaris equorum* (37%); dogs: *Dipylidium caninum* (20%), *Trichuris trichiura* (9%) and *Toxocara canis* (25%). In domestic guinea pigs: *Strongylus* spp (6.2*%)*; in cats: *Toxocara cati* (44%); birds cysts of *Eimeria* spp (15%) and cattle (50%) were positive for *Trichostrongylus* spp, *Eimeria* spp. (Table 1, Fig 3).

**Table 1:** Distribution of intestinal parasites of children and domestic animals found in the communities of Toche and Combia—Valle del Cauca–Colombia.

	Prevalence mean	Prevalence IC 95% ILPI-SLPI
Children		
All gastrointestinal parasites	60% (SD±9,6%)	41% - 78%
*Endolimax nana*	24% (SD±8,4%)	9,8% - 42%
*Iodamoeba buetschlii*	16% (SD±7%)	4,7% - 32%
*Entamoeba coli*	35% (SD±9%)	18% - 55%
*Giardia lamblia*	12% (SD±6%)	2,7%-27%
Dogs—*Canis lupus familiaris*		
*Dipylidium caninum*	20% (SD±8.7%)	6% - 39%
*Toxocara canis*	25% (SD±9%)	9% - 46%
*Trichuris trichiura*	9% (SD±6,5%)	1,3% - 26%
Cats—*Felis catus*		
*Toxocara cati*	44% (SD±15,7%)	16% - 75%
Equidae		
*Parascaris equorum*	37% (SD±16%)	10% - 71%
*Strongylus* spp	37% (SD±16%)	10% - 71%
*Strongylus + Parascaris*	37% (SD±16%)	10% - 71%
Birds—*G*. *gallus domesticus*		
*Eimeria* spp	15% (SD±8%)	3,4% - 33%
Cattle—*Bos taurus*		
*Trychostrongylus* spp, *Eimeria* spp	50% (SD±14%)	23% - 77%

**Table 2:** Samples analyzed for intestinal parasites from children located in Rural High Mountains of Toche and Combia—Valle del Cauca–Colombia.

Sample number	Municipality	Age/year	Sex	Parasite Diagnostic
1	Combia	2	Female	Negative
2	Combia	9	Female	*Endolimax nana*
3	Combia	7	Female	*Entamoeba coli*
4	Combia	9	Female	*Entamoeba coli* + *I*. *buetschlii*
8	Combia	0,4	Female	Negative
9	Combia	6	Female	*Giardia lamblia*
10	Combia	2	Female	*Giardia lamblia*
12	Combia	2	Female	*I*. *buetschlii*
15	Combia	13	Female	*I*. *buetschlii + Entamoeba coli*
5	Combia	6	Male	*Endolimax nana*
6	Combia	10	Male	*Endolimax nana*
7	Combia	11	Male	*Entamoeba coli + Endolimax nana*
11	Combia	1	Male	*Giardia lamblia*
13	Combia	9	Male	Negative
14	Combia	14	Male	*Entamoeba coli*
16	Combia	2	Male	*Entamoeba coli*
17	Combia	8	Male	Negative
1	Toche	3	Female	*Giardia lamblia*
2	Toche	0,3	Female	Negative
4	Toche	7	Female	*Endolimax nana* + *I*. *buetschlii*
5	Toche	0,3	Female	*Giardia lamblia* + *I*. *buetschlii*
3	Toche	10	Male	Negative
6	Toche	12	Male	Negative

In the interviews, it was found that the population was made up of an average of 2,5 people per household and the response rate was above 95%. 90,9% among them belong to a socio-economic stratum one (low-low); However, this level of stratification is unknown by most of the population of Toche (26%) and Combia (33%) (Table 3).

**Table 3:** Sociodemographic characteristics, personal hygiene practices, and symptoms of children with intestinal Pathogenic Parasites (PP) and Non-Pathogenic Parasites (NPP).

Variable/category	Negative	Positive	Odds ratio	SD	IP95%
**Gender** Female	3	9	6,72	9,36	0,66–28,01
Male	6	5			
**District** Combia	6	11	3,39	5,42	0,25–15,03
Toche	3	3			
**Child’s school year** Primary	4	7	0,33	0,47	0,006–1,51
Secondary	1	1			
**Family income** **≥**828,000 COP	2	3	2,46	15,39	0,12–10,43
<828,000 COP	7	11			
**House floor** Concrete	8	7	0,25	0,39	0,0037–1,2
Wood	1	5			
**Source of water** Aqueduct sidewalk	2	1	0,57	1,43	0,004–3,55
Head of river	7	13			
**Parasite therapy** Self-medication	0	2	6,60+24	1,2+26	26,23–2,2+24
Medical treatment	3	6			

All children are under the care of their biological mother. Their level of schooling was distributed as follows: incomplete primary (38,6%), complete primary (22,7%), complete secondary (9,1%), incomplete secondary (15,9%). 11,4% of parents did not show any level of education. 67,6% of those interviewed belong to the subsidized social security scheme and 20,6% of inhabitants are not aware of the benefits offered by the government. In general, the population earns less than the Colombian minimum wage, (236 dollars), which implies that for 66% of this group money is insufficient to cover all of their household expenses; therefore, 34,8% among them have struggled financially in order to purchase food. However, only 6,8% of children reported having starved.

Regarding the analysis of housing and sanitary hygienic aspects, we concluded that 75% of the participants do not have garbage dumps near their house since they have a garbage collection service provided by the municipality; and 25% must resort to the creation of areas for waste disposal due to its isolated location. About the type of housing, most of them live on concrete floor (59,1%), table (27,3%), land (9,1%) and tile (4,5%). The water supplied to these populations for cooking comes from natural water sources (59,1%); from the head of river (27,3%) and the village piped water supply (13,6%).

People in the area do not usually boil water for consumption (65,5%), have septic tanks or sewers (59,1%) and although the population reports washing their hands before eating (93,2%), children have a high exposure to soil and/or land during their nurture and feeding period (69%).

Only 14,3% of the population do not wash their hands before eating and 70,5% of the parents do not frequently deworm their children. Those who do deworm resort to medication provided by mobile health brigades (78,6%); however, there is a margin of self-medication of 21,4%, which directly or indirectly affects the reports of children with abdominal pain and diarrhea (78,2%).

The measures of association with the highest odds ratio (OR) are related with the gender OR 6,72 [IP95% = 0,66–28,1], district of (Combia and/or Toche), OR 3,39 [IP95% = 0,25–15,03] and family incomes OR 2,46 [IP95% = 0,12–10,43]; However, there is no significant risk in relation to other variables OR with similar IP (Table 3).

**Fig 4:**
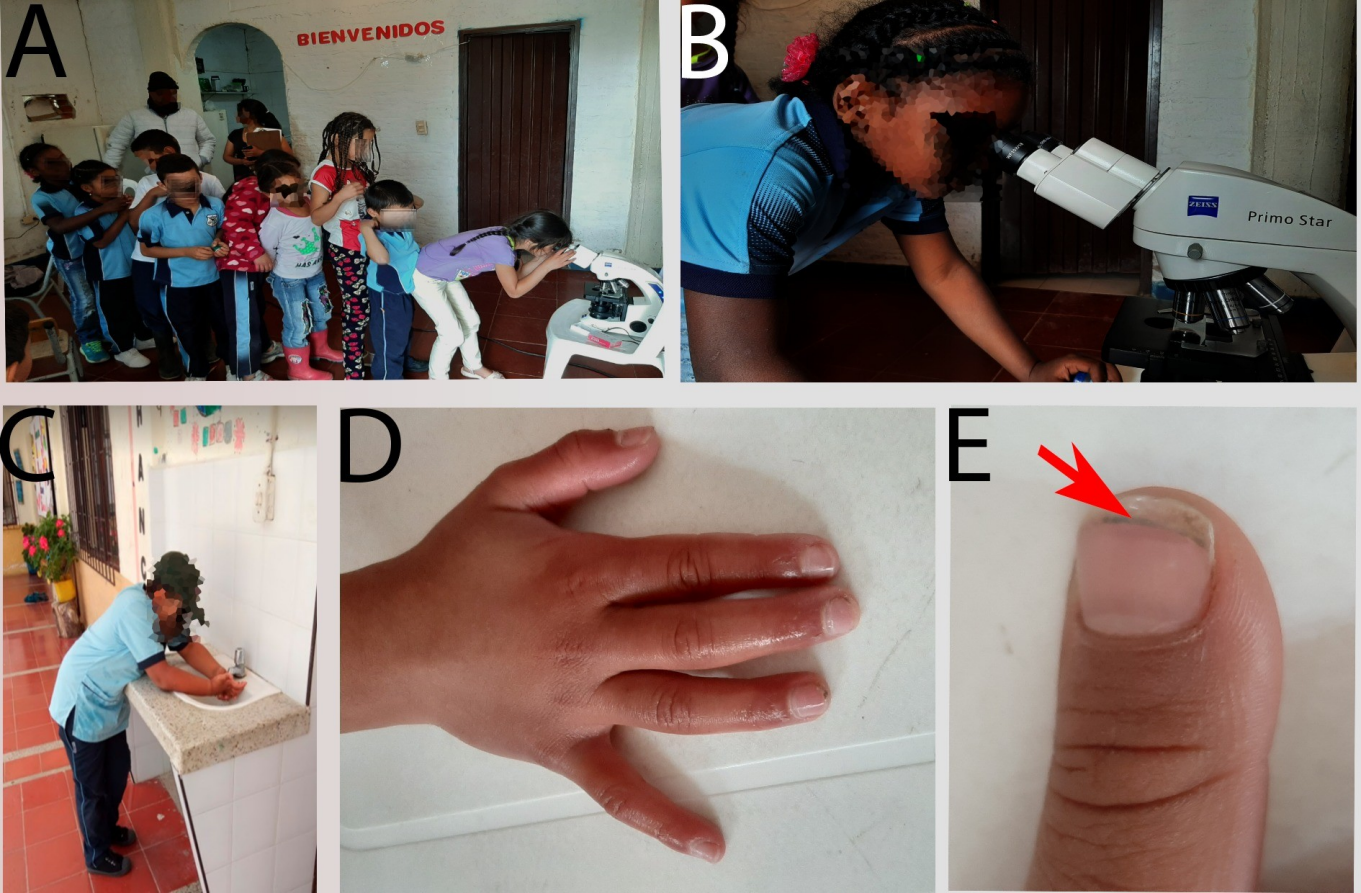
Educational practices to prevent parasites. Light microscopy as strategy to educate children about parasites and routes of transmission in the village of Teatino-Rita Sabogal School and Combia-Atanasio Girardot School (A,B). Campaign wash your hands with soap and water before eating and after leaving the toilet. The red arrow shows a dirty nail after three cleaning treatments (C,D,E).

In children under 6 years of age, it was found that the prevalence of parasites is 60% and the probability (OR 2,3) that children with contact with pets have a parasitic infection than those who do not have contact with animals. The probability that the intestinal damage reported in children is caused by parasites is low (OR 1,3), but the risk of infection may be even higher considering that the animal population have neither preventive or deworming health plans, nor veterinary assistance. Deworming and vaccination plans only apply to the bovine population. In this study, to contribute to the improvement of the quality of life of these communities, deworming plans for endoparasites and ectoparasites were carried out in canines and felines. This was linked to an educational plan for children and adults with an emphasis on a teaching campaign on personal hygiene and in order to raise awareness through the macroscopic and microscopic visualization of parasites (roundworms, tapeworms, flatworms and protozoa) about the importance of drinking potable water, and consuming clean food as the best way to prevent parasitic infections (Fig 4).

## Discussion

The districts of Combia and Toche have a population of 353 and 406 inhabitants, respectively. It is mostly composed of older adults (50%) and a children population of 50 individuals (18,7%) between 1 and 12 years old [26]. They are vulnerable populations, with low-income levels, (less than 1 dollar per hour), who have suffered violence and forced displacement. Due to this, only 25% of the total population resides in these rural communities [23]. The lack of job opportunities and the relocation of families in the urban area of Palmira results in a low child (< 6 years old) population (n = 23). In this study, the parasitic frequency in children was 60% lower than in indigenous children (84%), as reported previously [27]; Similarly, the presence of Giardia cysts (12%) was also less than the prevalence found in populations of children studied after natural disasters (60,4%) [11].

The children of Combia and Toche consume water without adequate treatment, from a common source of infection (school tap water). They do not live under the same roof, nor do there exist a high degree of overcrowding that allows a propagation factor, as presented in communal shelters as result of earthquakes; However, it should be noted that farms located in the upper part of the schools and homes represent a risk factors in the spread of Cryptosporidiosis, Giardiosis and other parasitic diseases.

Regarding the presence of *Endolimax nana* and *Iodamoeba buetschlii*, its importance is minor, although they are indicators of bad hygiene habits and fecal contamination of food [28]. In Colombia, frequencies of Giardiasis have been reported ranging from 0,1–62% and Cryptosporidiosis between 0,1–72%, varying between diagnostic methods, population type and immune status of individuals [29,30]. Interestingly, children from urban areas of Cauca Colombia were reported with a prevalence of *Blastocystis* (39,22%), *G*. *duodenalis* (10,59%), and *Cryptosporidium* spp (9,8%), Entamoeba complex 0,78% using quantitative real-time PCR (qPCR) [30]. In this study, Cryptosporidiosis and Blastocystosis was not observed in children, but its presence can be ruled out with sufficient accuracy with more sensitive techniques [30].

*Entamoeba coli* is a non-pathogenic amoeba. All visualizations were from cystic states, which measure 10–35 μm with 8–16 nuclei and central cariosome. In this study a significant percentage (15,6%) were found. *E*. *coli*, is part of the Entamoeba genus which includes seven species: *E*. *histolytica*, *E*. *dispar*, *E*. *moshkovskii*, *E*. *bangladeshi*, *E*. *poleki*, *E*. *coli* and *E*. *hartmanni*. *E*. *histolytica*, *E*. *dispar* and *E*. *moshkovskii*. These species are considered morphologically identical but genetically distinct; however, traditional methods do not allow differentiation, so they are reported as a complex. In humans, the Entamoeba complex is related to *E*. *dispar*, *E*. *histolytica* and *E*. *moshkovskii*, and it can only be differentiated by PCR. *E*. *dispar* is not pathogenic, but *E*. *histolytica* is; and the pathogenicity of *E*. *moshkovskii* is controversial [30]. In Colombia, all three species have been detected in asymptomatic children with a prevalence of 15% [31].

*Endolimax nana*, *Iodamoeba buetschlii*, *Entamoeba coli*, *Entamoeba hartmanni* are considered commensal species, so the founding of *Endolimax nana* (24%), *Iodamoeba buetschlii* (16%) are frequent and related to *Blastocystis sp*. (28,2%), *Endolimax nana* (14,9%), and *Giardia duodenalis* (11%) with similar frequencies as reported in this study. It is important to add that guard association with rotavirus and cryptosporidium is frequent [32]. The presence of *Dipylidium caninum* (12%) found in this study in dogs has been previously reported as a zoonosis in children [33], but no cases associated with the child population were found in these communities. *Trichuris trichiura* and *Ascaris lumbricoides* are high-presentation helminths in children (12,3% and 5,15%, respectively) [4]. In this study, *Trichuris trichiura* was found in dogs (9%), as well as *Toxocara canis* (25%). Although no eggs were found in the feces of children associated with this nematode, studies in Colombia have reported prevalence of 8,6% by stool test of canids (28) and 47,5% by serology in children [34].

Studies in other territories of Colombia have reported a prevalence of Uncinarias (20,6%) in canines (*Canis lupus familiaris*), *Toxocara canis* (8,6%), *Strongyloides* spp. (2,9%), *Entamoeba* spp. (21,1%), *Blastocystis* spp. (18,3%) and *Giardia* spp. (16%) (28); at this point, no evidence of the presence of *Uncynarias*, *Strongyloides* or *Blastocystis* spp was identified.

Other studies have reported *Giardia canis* (31,1%), *Isospora* spp (31,1%), the roundworm *Toxocara canis* (12,6%), the tapeworm *Dipylidium caninum* (7,6%), *Ancylostoma caninum* (4.2%) and *Cryptosporidium* spp (2,5%) [35], but under our methods we did not find traces of *Isospora* spp, *Ancylostoma* spp and *Cryptosporidium* spp. We hypothesize that the reason of which we did not find animals parasites on children (zoonosis) and children parasites in animals (anthropozoonosis) is because in these rural communities, children have less contact with cats and dogs, reducing the risk of transmission. This may be explained by the fact that there are no communal children parks or rural dog parks, which act as a source of transmission for canine parasites, including some with zoonotic potential such as *Toxocara* spp. Avoiding the children’s playgrounds which is one of the most frequent factor of contamination in other countries [36]. In the case of cats, they live outside of homes and behave as feral animals, that is, as an un-owned domestic cat that lives outdoors and avoids human contact. These animals catch prey, eat raw meat, do not have veterinary assistance and are not dewormed. They also do not have contact with children, which is a different scenario from urban areas and other reports, but it is still required to monitor these populations at the high mountain level and other interactions [37], as well as the capacity of these animals to spread parasitic diseases to wildlife species.

Although parasite diagnosis using microscopy has low sensitivity for the identification of intestinal protozoa compared with qPCR [30], microscopy remains the cornerstone of parasitological diagnostics, especially in the field and low-resource settings, and provides epidemiological assessment of parasite burden. Nevertheless, increased use and availability of point-of-care tests and molecular assays in modern era allow more rapid and accurate diagnoses and increased sensitivity in the identification of parasitic infections [38]. We know that DNA-based tools are increasingly being used for the diagnosis of intestinal worm infections in both clinical and research laboratories. Although recovering DNA from intestinal worm eggs in stool remains a challenge since this DNA is protected by a very rigid eggshell [39], *G*. *intestinalis*, *E*. *dispar*, *D*. *fragilis* and *Cryptosporidium* spp) have been successfully analyzed by real-time PCR and there are no differences in detection between the extended microscopy method and real-time PCR [40].

Future studies are necessary in order to analyze water sources, contaminated vegetables grown in rural high mountain areas, hands of food handlers (housewives) and drinking water used in animal and human food as well as to understand the One health approach considering that optimal health of humans is associated with the well-being of animals and the environment [41].

Finally, enormous efforts are required in vulnerable communities and rural areas of Colombia in order to prevent parasitic diseases. Both human medical assistance and veterinary services through preventive medicine are the most powerful tools in order to reduce the parasites frequency as well as to develop an effective and consistent plan of animal deworming which could improve the quality of health of these populations.

### Limitations of the study

The study has three important limitations that need to be considered when interpreting the results. Firstly, Combia and Toche are districts with a temporal or itinerant population; secondly, some areas were difficult to access and are located in distant territories; and finally, some people were reluctant to participate in order to protect their territories and to avoid political intervention. Additionally, in spite of having sampled all units (children), the sample size is too small to find some relationship or differences by statistical power. Therefore, is it possible to incur in errors (such as not find the differences where there are). Nevertheless, it is not reasonable to discard its hypothetical effects. On the contrary, this points to the importance of epidemiological approach considering a broad study population with hierarchical sampling design.

## Conclusions

Children under 6 years of age are more likely (OR 2,3) to get parasitic infection. An adequate education plan, at the school level, as well as a preventive anti parasitic plan will reduce the risks associated to parasite transmission. The results found were the basis for guiding an appropriate medical intervention in these communities and show the need for a greater government intervention.
